# Emergence and Polyclonal Dissemination of OXA-244–Producing *Escherichia coli*, France

**DOI:** 10.3201/eid2704.204459

**Published:** 2021-04

**Authors:** Cecile Emeraud, Delphine Girlich, Rémy A. Bonnin, Agnès B. Jousset, Thierry Naas, Laurent Dortet

**Affiliations:** INSERM, University Paris-Saclay, Le Kremlin-Bicêtre, France (C. Emeraud, D. Girlich, R.A. Bonnin, A.B. Joussett, T. Naas, L. Dortet);; Bicêtre Hospital, Le Kremlin-Bicêtre (C. Emeraud, A.B. Jousset, T. Naas, L. Dortet);; French National Reference Center for Antibiotic Resistance, Le Kremlin-Bicêtre (C. Emeraud, R.A. Bonnin, A.B. Jousset, T. Naas, L. Dortet)

**Keywords:** carbapenemase, OXA-48–like, Enterobacteriaceae, clones, whole-genome sequencing, next-generation sequencing, MLST, antimicrobial resistance, Escherichia coli, AMR, bacteria, France, carbapenemase-producing Enterobacterales

## Abstract

Since 2016, OXA-244–producing *Escherichia coli* has been increasingly isolated in France. We sequenced 97 OXA-244–producing *E. coli* isolates and found a wide diversity of sequence types and a high prevalence of sequence type 38. Long-read sequencing demonstrated the chromosomal location of *bla*_OXA-244_ inside the entire or truncated Tn*51098*.

Carbapenems are the last line antimicrobial drugs for treating infections caused by multidrug-resistant *Enterobacterales*. The global dissemination of carbapenemase-producing *Enterobacterales* (CPE; formerly known as carbapenemase-producing *Enterobacteriaceae*) pose a serious threat to public health (*1*). Oxacillin (OXA) 244, a single amino-acid variant of OXA-48 (Arg-222-Gly) (*2*), is an emerging carbapenemase variant in several countries in Europe (*3*–*7*). During 2013–2019, the French National Reference Center received a continuously increasing number of OXA-244–producing isolates for antimicrobial resistance (AMR) testing. OXA-244–producing isolates increased from 0 in 2012 to 72 in 2019. In France, OXA-244–producing *Enterobacteriaceae* represent 2.4% of all CPE and represented 3.4% of OXA-48–like producing CPE in 2019 (*8*). In addition, this tendency might represent only a fraction of OXA-244–producing *Enterobacteriaceae* because this variant is difficult to detect on CPE screening media due to the low hydrolytic activity of this carbapenemase (*8*). OXA-244 is found mainly in *Escherichia coli* isolates (*6*). The *bla*_OXA-244_ gene is described in only 1 type of transposon, Tn*51098*, a 21.9-kb IS*1R*-based composite transposon that includes a truncated Tn*1999.2* (ΔTn*1999.2*) and a fragment of the archetypal IncL *bla*_OXA-48_–carrying plasmid, pOXA-48 (*2*). 

Previous studies analyzed only a limited number of OXA-244–producing *E. coli* of an epidemic clone belonging to sequence type (ST) 38 that spread in countries in Europe (*3*,*5*,*7*,*9*). More data on the epidemiology and genetics of OXA-244 are required to understand its spread in Europe. We used whole-genome sequencing (WGS) to characterize the epidemiology of OXA-244–producing *E. coli* circulating in France during 2016–2019.

## The Study

**Figure 1:**
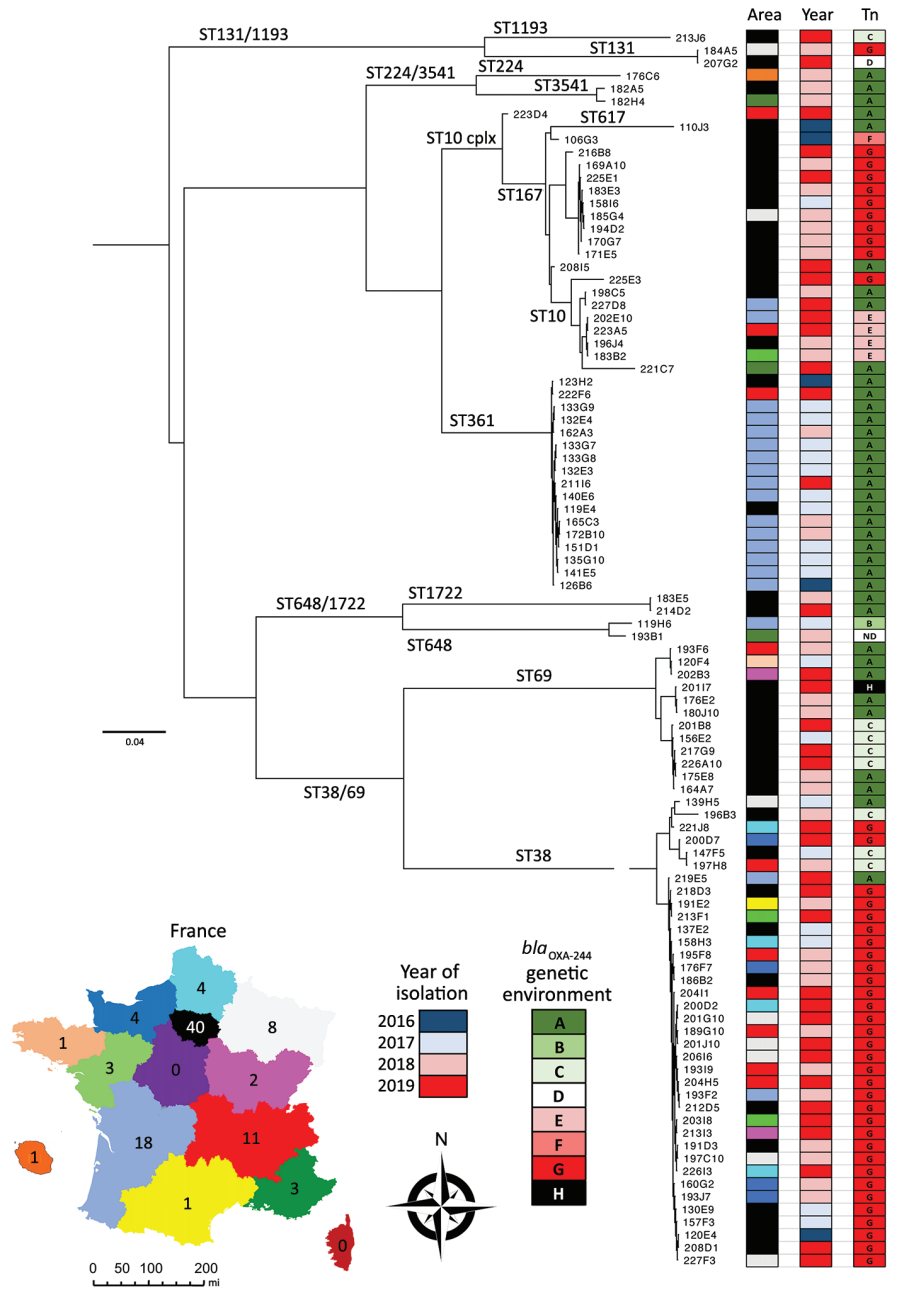
Phylogenetic relationship and geographic distribution of the 97 OXA-244–producing *Escherichia coli* isolates recovered in France, 2016–2019. Inset map shows regions of France; colors correspond to areas from which OXA-244–producing *E. coli* isolates were collected. The phylogenetic tree was constructed using CSIPhylogeny version 1.4 (https://cge.cbs.dtu.dk/services/CSIPhylogeny). Scale bar indicates nucleotide substitutions per site. ND, not detected; OXA, oxacillin; ST, sequence type.

During 2016–2019, the French National Reference Center identified 97 OXA-244–producing *E. coli* isolates. We performed WGS on all isolates by using the HiSeq (Illumina Inc., https://www.illumina.com) sequencing platform (GenBank accession nos. in Appendix 1 Table). We performed in silico multilocus sequence typing (MLST) by using the MLST 2.0 server (https://cge.cbs.dtu.dk/services/MLST). We identified 12 different sequence types (STs); the 5 most prevalent were ST38 (n = 37), ST361 (n = 17), ST69 (n = 12), ST167 (n = 11), and ST10 (n = 8) (Figure 1). Among OXA-244–producing *E. coli* isolates, the prevalence of ST38 rose from 12% in 2016 and to 47% in 2019.

On all 97 genomes of OXA-244–producing *E. coli*, we used a core genome single-nucleotide polymorphism (SNP)–based approach to create a phylogenetic tree by using CSIPhylogeny (https://cge.cbs.dtu.dk/services/CSIPhylogeny). To identify clades within STs, we performed a nested phylogenetic analysis with isolates of each ST to construct a SNP matrix. Isolates within the same clade would be highly suggestive of patient-to-patient cross-transmission of the same strain. We considered 2 strains to be part of the same clade if they were separated by <100 SNPs along their common genome (Appendix 2 Figure). We identified large clades corresponding to clonal dissemination of a single strain in the same area, including 5/12 isolates of ST167 and 15/17 isolates of ST361. We identified <7 different clades of ST38, and most (30/37) isolates belonged to the same clade (Appendix 2 Figure). However, these 30 ST38 isolates were collected in 9 different areas of France and 25 were isolated during 2018–2019 (Figure 1).

Because assembly of regions with repeated sequences was difficult with Illumina WGS data, we sequenced some isolates by using long read nanopore technology by using a MinIon (Oxford Nanopore, https://nanoporetech.com) sequencer (*10*). We performed WGS on 3 isolates belonging to the most prevalent STs: isolate 119E4 (ST361), isolate 120E4 (ST38), and isolate 156E2 (ST69) (Appendix 1 Table). We found a chromosomal localization of *bla*_OXA-244_ gene in the 3 isolates. By combining data obtained by both WGS technologies, we reconstructed the different genetic environments of *bla*_OXA-244_ gene and annotated the assembled sequences by using CLC Genomics Workbench version 12.0 software (QIAGEN, https://www.qiagen.com). 

**Figure 2:**
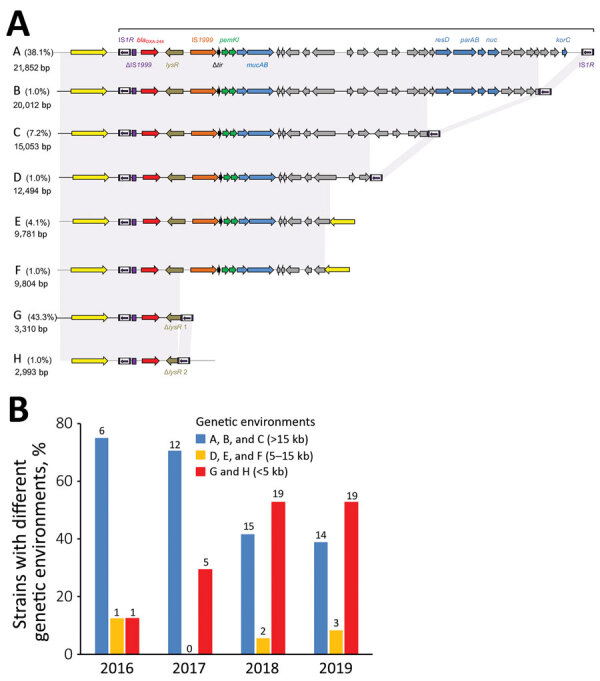
Genetic relationship and environment of *bla*_OXA-244_ genes identified in *Escherichia coli* isolates collected during 2016–2019, France. A) Schematic representation of the close genetic context of *bla*_OXA-244_ genes (A–H) identified in *E. coli*. Arrows indicate direction of the genes. Yellow indicates chromosomal genes; purple indicates mobile elements; red indicates β-lactamase genes; green indicates toxin–antitoxin genes; blue indicates genes with known function; and gray indicates hypothetical proteins. B) The relationship between the close genetic environment of *bla*_OXA-244_ gene and the year of isolation. OXA, oxacillin.

We detected 8 different genetic environments in our collection (Figure 2, panel A). Among the 97 *E. coli* isolates, 37 (38.1%) possessed the *bla*_OXA-244_ gene in Tn*51098*, a previously described transposon (*2*,*11*) (Figure 2, panel A). Among the other 60 (61.9%) isolates, we found *bla*_OXA-244_ in the shorter form of Tn*51098* (2,933–20,012 bp) (Figure 2, panel A). The *bla*_OXA-244_ gene still was systematically included in a truncated Tn*1999.2* (ΔTn*1999.2*), as described in *E. coli* VAL (*2*). For 44.3% of isolates, the remnant Tn*51098* was reduced in size (42 isolates with genetic environment G and 1 with genetic environment H) (Figure 2, panel A). We noted, the *bla*_OXA-244_ gene was included in a ΔTn*1999.2* where the *lysR* gene was truncated by the IS*1R* element. Of the 42 isolates sharing the genetic environment G, 32 (76.1%) belonged to ST38. By separating the type of genetic environment according to the date of isolation, we noticed that the short forms were isolated during 2018–2019 (38/44 strains, 86%) (Figure 2, panel B).

## Discussion

Dissemination of ST38 OXA-244–producing *E. coli* has been observed in many countries in Europe (*3*–*7*) and a few other countries around the world (*12*,*13*). However, most of these studies focused on ST38. Our results confirm the phenomenon of OXA-244–producing *E. coli* isolates in France because 38% of isolates in our study belonged to ST38. In addition, we observed an increased number of ST38 isolates during 2018–2019. Phylogenetic analysis identified a substantial clade inside ST38 (Appendix 2 Figure), but massive dissemination of this clone in France likely does not correspond to cross transmission of a single strain in different areas. The few SNP differences identified among ST38 isolates suggest this clade emerged recently. Accordingly, inside this compact ST38, the <100 SNP cutoff used to discriminate between 2 clades might be lowered because it was recently described for another high-risk clone, *Klebsiella pneumoniae* carbapenemase–producing *K. pneumoniae* ST258 (*14*).

The other common STs noted in our study are ST361, ST167, ST69 and ST10. In Europe, OXA-244–producing *E. coli* of ST69, ST167, and ST361 have been reported in Denmark (*6*), and ST69 and ST10 in Switzerland. Unlike what we observed with ST38, clones observed inside ST167 and ST361 mostly correlate with the same geographic area suggesting patient-to-patient cross-transmission.

As previously described for ST38 *E. coli* VAL (*2*), we demonstrated the chromosomal location of the *bla*_OXA-244_ gene in 3 isolates belonging to the 3 main STs, ST38, ST361, and ST69. The chromosomal localization of *bla*_OXA-244_ together with the intrinsic lower hydrolytic activity of OXA-244, compared with OXA-48, contribute to the difficulties in accurately detecting OXA-244–producing *E. coli* using classical screening media (*8*,*15*), suggesting a large underestimation of the real spread of OXA-244 producers.

In 2013, the *bla*_OXA-244_ gene initially was reported to be embedded in a 21,852-bp transposon Tn*51098*, which contains ΔTn*1999.2* (*2*). This structure still is present in 38.1% of OXA-244–producing *E. coli*. To our knowledge, Tn*51098* is the sole genetic structure reported for *bla*_OXA-244_. In our collection, *bla*_OXA-244_ was embedded in truncated forms of Tn*51098* in most isolates. Of note, in most (86.5%) ST38 OXA-244–producing *E. coli* the close genetic context of the *bla*_OXA-244_ gene was reduced to a small 3,310-bp fragment matching Tn*51098* and corresponding to a truncated form of the Tn*1999.2*. In addition, the most recently collected isolates possess short versions of the Tn*51098* compared with the isolates collected earlier (Figure 2, panel B). The effect on the clonal dissemination of this genome reduction around *bla*_OXA-244_ gene (e.g., better fitness) remains undetermined. Further analysis on the *bla*_OXA-244_ close genetic environment could elucidate the effects of this genome reduction.

Appendix 1 Global characteristics and NCBI accession numbers of OXA-244–producing *Escherichia coli* isolates.

Appendix 2 Phylogenetic tree of OXA-244–producing *Escherichia coli* in 5 major sequence types, France.
