# Supplementary material for: Emergence and Polyclonal Dissemination of OXA-244–Producing Escherichia coli, France
Source: Emerg Infect Dis. 2021 Apr;27(4):1206–10. doi: 10.3201/eid2704.204459 (PMC8007313; doi:10.3201/eid2704.204459)
Supplement: Appendix 2 — Phylogenetic tree of OXA-244–producing Escherichia coli in 5 major sequence types, France. [file 20-4459-Techapp-s2.pdf]

Article DOI: https://doi.org/10.3201/eid2704.204459

Emergence and Polyclonal Dissemination of OXA-244–Producing *Escherichia coli*, France

Appendix 2

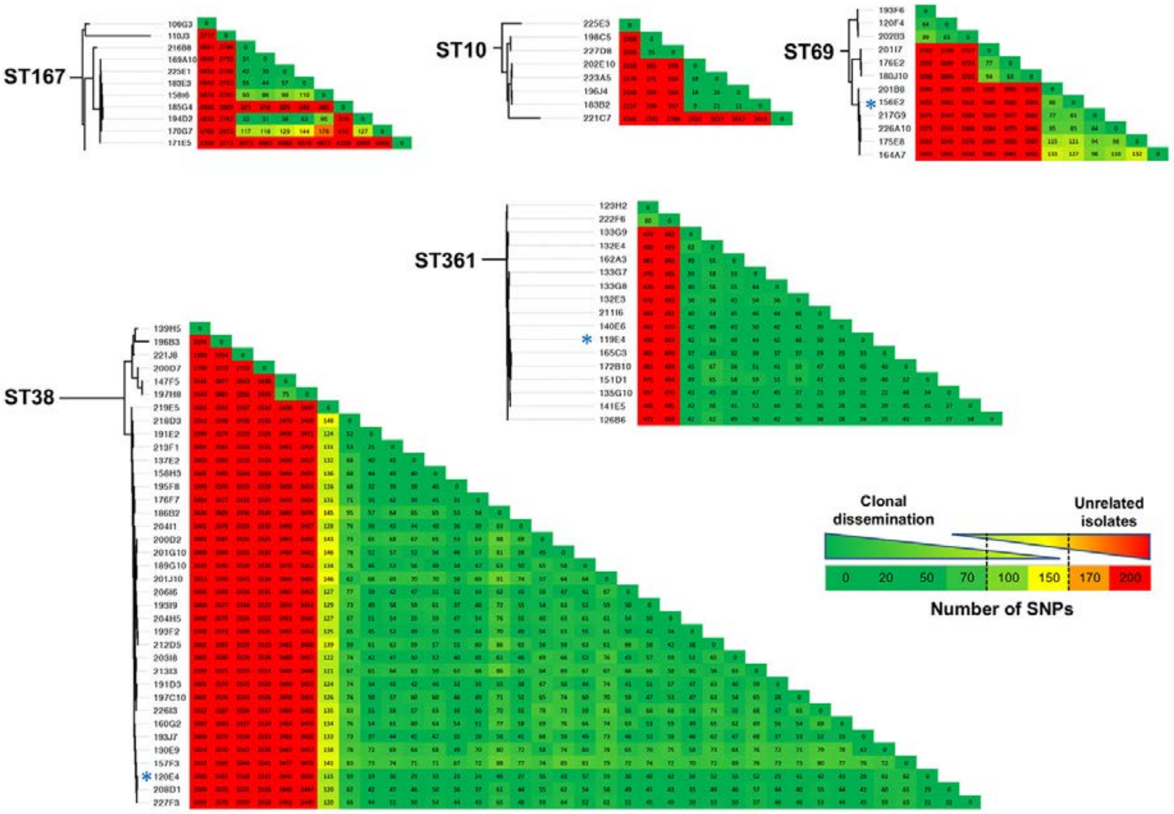

Appendix Figure. Phylogenetic trees with SNP matrixes for OXA-244–producing *Escherichia coli* belonging to the 5 major sequence types: ST38, ST69, ST361, ST167, and ST10. Blues asterisks correspond to OXA-244–producing *E. coli* isolates sequenced by both HiSeq (Illumina, https://www.illumina.com) and MinION (Oxford Nanopore, https://nanoporetech.com). SNP, single-nucleotide polymorphism; ST, sequence type.
